# Announcement: World Hepatitis Day — June 28, 2017

**DOI:** 10.15585/mmwr.mm6629a5

**Published:** 2017-07-28

July 28, 2017 is World Hepatitis Day, an annual day of observance established by the World Health Organization to promote awareness and understanding of viral hepatitis. An estimated 325 million persons are infected with chronic hepatitis B virus (HBV) or hepatitis C virus (HCV) worldwide (*1*), and an estimated 1.3 million persons die from related causes annually (*1*). In June 2016, the World Health Assembly endorsed the Global Health Sector Strategy on Viral Hepatitis 2016–2021, which sets goals for the elimination of HBV and HCV as global health threats by 2030 and outlines the global actions needed to reach these goals (*2*). The theme of this year’s World Hepatitis Day is “Eliminate Hepatitis.”

This issue of *MMWR* includes a report on progress toward achieving HCV elimination in the nation of Georgia, which in April 2015, became the first country in the world to launch such a program. Georgia has set an ambitious goal of 90% reduction in HCV prevalence by 2020. Documenting Georgia’s progress, challenges, and strategies to address the challenges can inform global HCV elimination actions. Additional information and resources are available at https://www.cdc.gov/hepatitis.
